# Reading Comprehension Tests for Children: Test Equating and Specific Age-Interval Reports

**DOI:** 10.3389/fpsyg.2021.662192

**Published:** 2021-09-10

**Authors:** Patrícia Silva Lúcio, Fausto Coutinho Lourenço, Hugo Cogo-Moreira, Deborah Bandalos, Carolina Alves Ferreira de Carvalho, Adriana de Souza Batista Kida, Clara Regina Brandão de Ávila

**Affiliations:** ^1^Department of Psychology and Psychoanalysis, State University of Londrina, Londrina, Brazil; ^2^Department of Psychobiology, Federal University of São Paulo, São Paulo, Brazil; ^3^Faculty of Teacher Education and Languages, Østfold University College, Halden, Norway; ^4^Assessment and Measurement PhD Program, James Madison University, Harrisonburg, VA, United States; ^5^Department of Speech-Language Pathology and Audiology, Federal University of São Paulo, São Paulo, Brazil

**Keywords:** reading comprehension, equating, concurrent calibration, anchor items, item response theory

## Abstract

Equating is used to directly compare alternate forms of tests. We describe the equating of two alternative forms of a reading comprehension test for Brazilian children (2nd to 5th grade), Form A (*n* = 427) and Form B (*n* = 321). We employed non-equivalent random groups design with internal anchor items. Local independence was attested *via* standardized residual Pearson's bivariate correlation. First, from 176 items, we selected 42 in each form (33 unique and 9 in common) using 2PL model, a one-dimensional item response theory (IRT) model. Using the equateIRT package for R, the anchor items were used to link both forms. Linking coefficients were estimated under two different methods (Haebara and Stocking–Lord), resulting in scores equating by two methods: observed score equating (OSE) and true score equating (TSE). We provided reference-specific age-intervals for the sample. The final version was informative for a wide range of theta abilities. We concluded that the forms could be used interchangeably.

## Introduction

Reading comprehension is a complex skill that depends on different cognitive and linguistic abilities (such as word recognition and working memory) related to the integration of text content to the strategies, expectancies, and previous knowledge of the reader (Cain et al., 2004; Van den Broek et al., 2005). Such complexity may be at the core of typical problems that learners face to understand the passages, which may be reinforced by disturbances, such as dyslexia or attention difficulties (Snowling and Stackhouse, 2016).

Good and poor comprehenders differ from each other in their capacities of extracting evidence from contextual cues—inferential skills (Yuill and Oakhill, 1996; Paul and Elder, 2012)—as well as their capacities of creating a mental representation of the read text (Kintsch, 1998). Therefore, for the evaluation and diagnosis of reading comprehension difficulties, it is necessary to explore the local and/or global process by which the reader may fail in grasping the meaning of the text (Van Dijk and Kintsch, 1983). In other words, and following, Cain and Oakhill (1999), it is crucial to investigate the failures of the inferential process, which may involve both the propositional level (called text-connecting inferences) and the integration of the content of the text with the previous knowledge of the reader (i.e., gap-filling inferences)^1^.

One challenge in constructing tests for evaluating cognitive/linguistic abilities is conciliating, in a single measure, the points of view of construct representation and of nomothetic span. In a classical article, Whitley (1983) defines construct representation as the process of identifying the mechanisms underlying the task performance, which is obtained by task decomposition. In the case of reading comprehension, it represents the inferential processes described above for understanding the message of the text. Although the inferential process is essential for understanding the passage, it necessarily does not interfere with the individual differences in the performance. It is a question of nomothetic span which, in the terms of Whitley, refers to the network of the relationship between the items of the test and a set of other measures. In other words, while construct representation is concerned with task variability, nomothetic span refers to subject variability. From the nomothetic point of view, it is important to keep interindividual variation (what can be achieved using a heterogenous sample of subjects or using items that inform about different strata of a latent trait).

The present study describes the process of equating alternative forms of a reading comprehension test for Brazilian children. As explained later (Methods section), both forms were idealized to allow for the interpretations arisen from construct representation and nomothetic span approaches. Creating alternative or parallel forms of an instrument is referred as the best way we can compare test scores across different sets of items that supposedly measure the same construct (American Educational Research Association et al., 2014). In a review, Oliveira et al. (2016) showed there are few standardized measures for evaluating reading comprehension among Brazilian children, and these lacked construct validation and reliable norms. The great majority of the instruments were not commercialized (they were available only in dissertations, chapter books, and papers) and none of the revised instruments presented item analysis. Since the review by Oliveira et al., this picture did not change a lot. Although two new instruments are now available, both lack information about the properties of the item. The “Anele” by Corso et al. (2020) presented a very small sample (100 students with 3–5 years of schooling) equally distributed in private and public schools (in Brazil, only 18% of students attend private schools). The authors reported a ceiling effect among children from private schools and the older children from public schools. There is information about internal consistency (alpha = 0.78) and interrater agreement (Kappa = 0.810), but validity studies are limited to age and socioeconomic differences and correlations with other instruments. The PROLEMLE (Cunha and Capellini, 2019) presented a wider sample (378 students from 3rd to 5th grades), but homogeneous in terms of the type of school (public) and from a convenience sampling. The authors reported temporal stability using McNemann's test between the first and the second occasions by item. Internal consistency varied from 0.63 to 0.69. Both Anele and PROCOMLE instruments are composed of multiple-choice questions (though the former additionally presents a free retelling). Finally, the instrument by Saraiva et al. (2020) cited by Oliveira et al. (2016) received a new edition in 2020. Albeit some improvements were observed in terms of construct description and scoring, it still does not present item analysis or validity and reliability reports. From the above, the effort for creating interchangeable forms of such an instrument is worthwhile.

The use of two equivalent forms of a test, especially when it comes to performance tests, is important for all purposes in which evaluation occurs in different sessions, for example, to compare change after treatment or to catch developmental changes. The great advantage of using equivalent forms instead of repeating the same form reduces the effects of the practice. Although alternate forms of a test share content coverage and procedures, they do not necessarily share their statistical proprieties (Urbina, 2014). It means that, in this last case, differences between occasions are more susceptible to measurement error or to differences in validity.

The success of equating depends intrinsically on the plan for data collection, which must be connected to the intended uses of the linked tests. A wide variety of research methods may be used (Angoff, 1971; Kolen and Brennan, 2014). Comparing the scores arisen from different assessments or putting them on a common scale has a long history in psychology and education (Kolen, 2004). Terms like calibration, linking, scaling, and equating are used in narrow or in broad senses, depending on the theoretical perspective adopted by the researcher. Linking refers to putting item scores on the same scale (Hambleton and Swaminathan, 1985) and, therefore, test information functions and test characteristic curves of both tests should be similar (McDonald, 1999). Strictly speaking, equating applies to tests that shares the same framework (domain and content) and the same specifications (such as formats, procedures, and rules for scoring), in such a way that they could be called as test forms (Kolen and Brennan, 2014). Equating forms depends on the demonstration that items of both tests present the same difficulty levels and content coverage, so that they can be used interchangeably (Kolen and Brennan, 2014; Bandalos, 2018). Kolen and Brennan (2014) defined equating as the statistical process used to adjust the differences in item difficulties in such a way that test scores that represent alternate forms can be directly compared to each other. For the purposes of this paper, we follow the definition of equating by Kolen and Brennan.

Despite the widespread use of IRT modeling in the educational and psychological research, reporting the equating of parallel test forms *via* IRT with real data seems to be scarce in the literature. We performed a nonsystematic review in PubMed and Science Direct (using the terms, “reading comprehension” and “equating” or “concurrent calibration”) and we found only 32 studies. From these, only nine were related to equating reading comprehension tasks with real data (Ree et al., 2003; Betts et al., 2009; von Davier, 2013; Liao et al., 2014; Dimitrov, 2016; Sandefur, 2018; Seo et al., 2018; Wagner et al., 2018; Rodrigues et al., 2020). In general, these studies present a wide diversity of approaches for reporting equating.

A great part of the research in the educational area used classic or observed scores approaches to equating (Ree et al., 2003; Betts et al., 2009; von Davier, 2013; Wagner et al., 2018). Most part of the studies that used IRT approach used Rasch models, which do not provide information about the discrimination of the items (Liao et al., 2014; Sandefur, 2018; Seo et al., 2018; Rodrigues et al., 2020). The exception is the study of Dimitrov (2016) that used 3PLM. The great majority of the revised studies are concerned with multiple-choice tests, with the exception being the studies by Betts et al. (2009) that equated a measure of fluency in reading and of Wagner et al. (2018) that used a mix of open-constructed and multiple-choice questions from Program for International Student Assessment (PISA). It is also remarkable that the studies generally focus on adult or youth population, with only two studies (Betts et al., 2009; Rodrigues et al., 2020) focusing on younger children.

In the present study, we report equating of two forms of a reading comprehension test for children using common (or anchor) items and random groups design. To overcome the limitations, multiple-choice tests format for evaluating active processing in reading comprehension (Ozuru et al., 2013), we used open-ended questions. We used different statistical approaches for equating. First, we tested for local independence of the items (which are embedded in texts) through bivariate standardized residual inspection (separately for each form); second, we reported the process of selecting items to calibrate the effect of anchor length (Kolen and Brennan, 2014), where items were selected from results taken from separate (one for each form) 2PL IRT analyzes. We used 2PL models because no guessing is expected for free-response questions and, differently from Rasch models, as they provide information about the index of discrimination of the items; third, we described IRT parameter linking with two different methods (namely, Haebara and Stocking–Lord) using the R equateIRT package. We used one of these methods to illustrate the conversion of one form to another; fourth, the observed-score equating (OSE) and IRT true-score equating (TSE) are reported using these two methods; finally, we presented referenced norms based on age-specific intervals for both forms, using the OSE method.

## Methods

### Ethical Statement

This study adheres to the ethical standards for research involving human being and received approval from the Ethical Committee (protocol number 38406/12). We evaluated only children whose parents provided informed, written consent.

### Sampling

Stratified random sampling was performed based on the 2008 school census for the city of Sao Paulo, Brazil (Instituto Nacional de Estudos e Pesquisas Educacionais Anísio Teixeira, 2009). At that time, São Paulo presented a population of more than 11,200,000, distributed in 32 district councils in five areas (North, South, East, West, and Center). The areas presented distinct population density, varying from 7,700 (West) to 16,400 (Center) inhabitants per km^2^. The West and the Center areas are very close to each other (and are territorially small in relation to the other areas); therefore we gathered them together, resulting in the following: North (9,400); South (12,400); East (13,100); and Center/West (8,300). These four areas are highly heterogenous in terms of socioeconomic status: The Center/West region, although the least populated (in relation to the other three), is richer. The South presents high levels of social inequalities (i.e., very rich and very poor people living together in territorially similar areas) and the other areas are formed by lower-middle class to poor areas.

The logistic of collecting data in a mega city with a big extension of area (1,500 km^2^) was taken in consideration when doing the sampling. For sampling, we decided drawing 20% of the 32 districts, totalizing 6.4 (what we converted into 7 districts). To account for the heterogeneity of the city, we considered the population density of the area and its territorial extension for drawing. As the North and Center/West regions presented the lowest population density, we drew one district from each. Although the South presented a lower population density than the East, it is a very heterogeneous region and with greater territory size. Therefore, we drew 3 districts from this region and 2 from the East. The councils were Santana (North), Pinheiros (Center/West), Penha and Aricanduva (East), and Vila Mariana, Campo Limpo, and Capela do Socorro (South).

Later, we computed the number of related schools in the seven selected districts (local, state, and private schools). From the total of 690 schools, we determined 3% for sampling (*n* = 21 schools). Based on the census, the proportions of schools according to the education system were defined as follows: 0.36, local (*n* = 7); 0.47, state (*n* = 10); and 0.17 (*n* = 4), private schools. Because the schools are not symmetrically distributed in the seven districts (i.e., private schools tend to be located in middle to high class neighborhoods, local schools to be dispersed in the peripheral areas, and state schools in the central areas of the city), the selection of schools did not consider the stratification by the district. As the statistical analysis would involve IRT models, we considered a total sample of about 800 participants appropriate for our purposes (Tsutakawa and Johnson, 1990). The last stratum considered was the school year, in which we equally divided the sample among the four strata (i.e., 2nd to 5th year), because the differences in the number of enrollments between the school grades seemed negligible (Instituto Nacional de Estudos e Pesquisas Educacionais Anísio Teixeira, 2009).

### Sample

This study considered the following exclusion criteria: absence of auditory or uncorrected visual disturbances; alleged neurological, behavioral, or cognitive impairments; complaints of specific learning difficulties; and history of school retention. The eligible children were identified by their teachers, who forwarded a letter describing the study (its aims, procedures, and measurements) and inviting the parents to provide a written consent for the participation of their children.

The children whose parents provided the written consent (*n* = 826) were screened for struggle in reading ability, because a minimum proficiency in decoding is required for performing the comprehension tasks. The screening task was a short text suitable for the school grade (ranging from 206 and 235 words), which should be read aloud by the child^2^. The time was registered at the beginning of the reading and, after 1 min, the examiner put a mark at the last word read. The rate of reading until this mark was the intake criterion. The cutoffs were 50, 66, 77, and 95, respectively for accurately read words for the 2nd to 5th year, respectively. The children who failed the task were withdrawn from the study. Therefore, 755 children were eligible for the study (57.6% girls) from local (37.1%), state (46.6%), and private schools (15.9%). The mean age of the children was 9.10 ± 1.02 years (age range = 6–12 years). Table 1 summarizes the characteristics of the children per group.

**Table 1:** Sample characterization per form of the test.

**Variable**	**Form A**	**Form B**
*n*	431	322
Mean age (S.D.)	9.1 (1.0)	9.1 (1.0)
Age (range)	7–11	6–12
Female (%)	57.0%	59.0%
Public school (%)	83.2%	85.0%
School year (%)		
2nd	27.2%	23.8%
3rd	24.4%	24.4%
4th	25.1%	25.9%
5th	23.3%	25.9%

Because of missing data, the comprehension tests were available for 748 children: 427 for Form A and 321 for Form B. Children were randomly assigned to Form A or Form B through coin flipping by the examiner before the beginning of the session. The frequency of children who completed Form A or B did not differ in age [*t*_(746)_ = 0.553, *p* = 0.580] or sex [$\chi_{\left(1\right)}^{2}$ = 0.318, *p* = 0.573].

### Equating Design

The method we used for equating was the common-item nonequivalent random group design, in which the participants were submitted to distinct forms of the instrument with some items applied to the total sample (the last design showed in Figure 1.1 of Kolen and Brennan, 2014). Hence, the children from the selected schools were randomly assigned to Form A or B. Additionally, all participants were exposed to a text and a set of related items to equate difficulty (anchor text items). The anchor items were used to compose both the raw scores and the final estimated theta scores of the examinees; therefore, these items were classified as internal to the scores (Kolen and Brennan, 2014).

### Instruments

The reading comprehension test that is currently in construction is comprised of two forms (Form A and Form B), each one composed of seven texts intended to be alternative forms. An additional passage and its items are common to both forms (which serve as an anchor). The passages are followed by open-ended questions (dichotomously scored) developed to evaluate different cognitive processes demanded to answer them correctly, covering, therefore, the construct representation of the task (Whitley, 1983). This was because poor comprehenders differ from typical readers in their difficulty in answering questions that demand different types of inferences, as well as their ability to draw conclusions from evidence or contextual clues (Yuill and Oakhill, 1996; Paul and Elder, 2012). These questions are described as follows: (1) literal that evaluates the retention of explicit information present in the text; (2) text-connection (TC) inferences that require the integration of implicit information present in the text to achieve coherence between different periods or phases; (3) gap-filling (GAP) that requires the use of background knowledge to fill the gaps of implicit or missing information in the text; and (4) situation model (SM), a kind of idiosyncratic mental representation of the situation expressed in the text (Kintsch, 1998; Cain and Oakhill, 1999).

Moreover, texts were created to follow progressive levels of difficulty, by adding the number of words and by reducing the readability across texts. As narrative texts are easier for readers (Kraal et al., 2018), only two expository texts were created for each form (the highest difficulty^3^ text, Text 7, with more words and lower readability, and Text 4, the middle difficulty text). Additionally, most texts presented middle levels of readability (avoiding very easy or very hard texts). This was performed to catch interindividual differences between the school grades, following the nomothetic principle (Whitley, 1983). Although Texts 4–7 were created to present the highest levels of difficulty of the instrument, the content of the passages deals with subjects that may be of general interest to the child and to the youth population (see Table 2, Note).

**Table 2:** Characterization of the texts and its questions (anchor, Form A, and Form B) before and after item selection.

**Text^**a**^**	**FK**	**Words**	**Original questions**					**Selected questions**				
			**(Classification)**					**(Classification)**				
			**LIT**	**TC**	**GAP**	**SM**	**Total**	**LIT**	**TC**	**GAP**	**SM**	**Total**
Anchor	52	192	1	6	1	1	9	1	6	1	1	9
1A	71	67	2	5	1	1	9	1	1	1	1	4
1B	85	79	2	9	2	1	14	1	1	1	1	4
2A	76	111	1	6	2	2	11	1	2	1	0	4
2B	61	101	3	4	6	1	14	1	2	1	0	4
3A	64	97	1	5	2	1	10	1	2	1	1	5
3B	58	116	3	5	4	2	13	1	2	1	1	5
4A	63	211	3	5	5	2	15	0	3	2	1	6
4B	62	165	1	10	3	2	16	0	3	2	1	6
5A	56	158	2	4	4	2	12	0	3	1	0	4
5B	57	223	1	9	3	0	13	0	3	1	0	4
6A	54	310	0	6	6	2	14	0	4	0	0	4
6B	51	210	2	12	0	0	14	0	4	0	0	4
7A	34	386	1	5	2	2	10	1	3	2	0	6
7B	40	322	1	8	2	0	11	1	3	2	0	6
Mean/Total^b^	58/63	158/ 165	24	99	43	19	185	9^c^	42^c^	17^c^	7^c^	75^c^

*Texts 4 and 7 of both forms are expository and all the other texts are narrative*.

*A, Form A; B, Form B; LIT, literal; TC, Text connecting; GAP, gap-filling; MS, situational model*.

*FK, Flesch-Kincaid Index. It evaluates readability based on the correlation between the average word sizes of the sentences. It gives a score between 1 (lowest readability score) and 100 (highest readability score). The adaptation of the FR to Portuguese was carried out by Martins et al. (1996). Formula: FR = 248,835 – [1,015 × (Number of words per sentence)] – [84.6 × (Number of syllables in the text/Number of words in the text)]. The classification of this index for Brazilian Portuguese is very easy (100–75); easy (50–75); Hard (25-50); and very hard (0–25)*.

a *Content subjects of the passages: Anchor = A bear runs away from his cage in the zoo; 1A = A girl plays in a garden; 1B = A boy gets a book as gift; 2A = A dog plays around; 2B = A girl is afraid of taking a vaccine; 3A = A ball falls in the neighbor yard; 3B = A boy cheats in the game with his friends; 4A = The anteater; 4B = The rufous hornero; 5A = A girl looks forward to the visit of Santa Claus; 5B = A boy is in a dark grove; 6A = The fisherman and his son go to work; 6B = A couple drawing water from the cistern; 7A = The origin of the pyramids; 7B = The origin of limestone rocks*.

b *The last line in this table presents mean values for FK and number of words and total for item's classification. Anchor item's values were not used for mean. The value before the bar/refers to mean for Form A and the after for Form B*.

c *The total considered the specific questions of both forms plus the common items (e.g., for LIT questions: 4 items in Form A; 4 items in Form B; 1 anchor item = 9 LIT questions)*.

The total test comprised 185 items, which were distributed as follows: 81 from Form A, 95 from Form B, and 9 from anchor items. Text types were narrative or expository and all were followed by 9–16 questions (see Table 2, Original Questions and Classification). The type of questions was not equally distributed in the texts because their elaboration depends on the context of the passage. Yet for this reason, the texts presented unequal initial pool of items (we created as much as questions were possible for testing this pool and selected the best and paired items). Nevertheless, the equivalence of the respective texts between the forms was considered in the item selection, as described later (i.e., the same number and type of questions in Texts 1A and 1B, 2A and 2B, and so forth). Table 3 presents two paired texts and their related questions (translated to English).

**Table 3:** Example of texts and related questions in each form (translation).

**Text 2—Form A**	**Text 2—Form B**
John opened the door. Buddy left the car, very excited. He was wagging his tail and running, looking at everything around him. There was so much news!	That was a different Saturday. Many children were waiting in line to be attended to. As soon as she arrived, Emma began to hear a chorus of cries. She was worried. From the room he was supposed to enter, some children came out sobbing, though others
Along the way, he smelled a lot of daisies and calla lilies, besides the grass. He went into the flowers, sniffing at the soil, until he smudged his muzzle in a puddle. Buddy stopped, and suddenly the mud moved. He saw a strange animal and ran away barking back to its owner. When him saw what it was, he caressed him and said:	seemed quiet Emma heard her name called and entered. There was a jar of strawberry lollipops on the table. The nurse handed one to each child. Emma lifted the sleeve of her shirt and gripped her mother's hand tightly. Her eyes closed and two tears streamed down her cheeks. But after feeling only a mince, she began to feel better
- It's just a frog …	
But Buddy continued barking, staring at the animal. John took its collar and leash. They went for a walk somewhere else	
1. For what reason John opened the door? (TC)	1. Where the children were waiting? (LIT)
2. What flowers Buddy smelled along the way? (TC)	2. How did the children know that was their turn to enter in? (TC)
3. What kind of puddle Buddy found? (TC)	3. **What there was on the table? (LIT)**
4. **Why did Buddy bark? (GAP)**	4. What did the nurse give to the children? (TC)
5. **Why did the mud move? (TC)**	5. Why did the nurse give lollipops to the children? (GAP)
6. Where was the strange animal that Buddy found? (TC)	6. Which was the flavor of the lollipop? (LIT)
7. **What was the strange animal that Buddy saw?** (TC)	7. **Why Emma was worried? (TC)**
8. Why did the Pingo's owner rub him? (TC)	8. Why did Emma lift her shirtsleeve? (GAP)
9. **What did John put on Buddy for a walk? (LIT)**	9. Why did Emma grip her mother's hand? (GAP)
10. It was raining or sunny on the day before? Why? (GAP)	10. Why did Emma feel a sting? (GAP)
11. Why did Buddy would do if he found a crab on the beach? Why? (SM)	11. **Why did some children look calm when leaving the room? (GAP)**
	12. When did Emma begin to feel better? (TC)
	13. **What did Emms won when she left? (TC)**
	14. What do you think Emma could say to the children that remained in line? (SM)

*LIT, literal; TC, Text connecting; GAP, gap-filling; SM, situation model. Selected questions are in bold*.

A pilot study (Lúcio et al., 2015) demonstrated age differences related to the type of questions, and the construct validity was attested (for each form separately) using 2PL one-dimensional models of item response theory (IRT). The test underwent psychometric inquiry using one-dimensional model and the items presented good inter-rater reliability (Lúcio et al., 2016), with mean Fleiss Kappa of 0.68 (Form A) and 0.80 (Form B), respectively.

### Procedures

Data collection was carried out from August to November 2012 that took place in the second semester of the academic years in Brazil. Children were individually tested by trained speech-language therapists in a quiet room at their own schools. Altogether with the reading comprehension test, they performed a set of tests that composed a battery, including decoding, oral comprehension, working memory, and rapid automatized naming. The sessions were in a total of 4, each one intercalated with two texts of reading comprehension, one cognitive task, and a play activity not related to the research. In general, the sessions lasted for 45 min each. For this study, we described the procedures related to the comprehension test.

The examiner asked the children to read the short passages the way they were used to comprehend (e.g., reading aloud or silently)^4^. The children were instructed to pay attention and try to understand the meaning because some questions about the text would be asked right after the reading. The test was not timed, and the examiner orally provided the questions. The questions were orally given to avoid the effect of the developmental differences in spelling performance. The texts remained with the examinees while the questions were presented. The answers were recorded for posterior scoring. All the questions were scored with 0 (wrong) or 1 (right) points. Responses that were incorrect according to an answer key and no-responses were considered incorrect.

### Statistical Analysis

For item selection and equating, one-dimensional models were considered for reading comprehension (i.e., all the items of each form running under a general factor). We used different steps to equate the two forms. Firstly, the local independence of the forms was checked for each form separately *via* bivariate Pearson standardized residuals (z-score; Haberman, 1973; Agresti, 2019). The idea underlying this evaluation was that items within the same text might show violations in the local independence when compared to the other text items. For the bivariate tables, the standardized Pearson residuals were computed using Equation 1, where O and E are the observed and expected (model estimated) quantities for a pattern in the categorical data (i.e., correct and incorrect answers) and *n* is the sample size. Traditionally, standardized residual z-scores exceeding |1.96| would indicate violations of local independence. However, in our context, where the number of items per form is 81 and 95, to decrease the false discovery rate (i.e., Type I error), we increased the cutoff based on the Bonferroni correction. Then, the significance level of 0.05 would be divided by 81 for Form A and 95 for Form B resulting in a new adjusted level of significance of 0.0006 for Form A (corresponding to a z-score of around |3.42|) and 0.0005 for Form B (corresponding to a z-score of around |3.47|).


(1) $$\begin{array}{l} \frac{O-E}{\sqrt{E}*\sqrt{\frac{1-E}{n}}} \end{array}$$

Second, we reduced the number of items in each form to comply with the rule-of-thumb suggested by Kolen and Brennan (2014), where the common-item set should contain at least 20% of as many items as the full test^5^. Considering we have 9 available anchor items, a maximum of 36 items should be selected for each form. We selected 4–6 items in each text, following the criteria described hereafter (Table 2).

The item selection was based on two criteria: first, the classification by same number and questions between corresponding texts (Text 1, Text 2, and so on). Second, we gave preference to items with a higher discrimination index (*a* parameter) and heterogeneous in terms of difficulty (*b* parameter) within each text. Discrimination and difficulty parameters were estimated based on the 81 items from Form A and 95 from Form B, separately. In this step, we used *Mplus* version 8.0 (Muthén and Muthén, 1998–2017) and all standard errors for the estimates were adjusted for the multilevel design. To that aim, we used the COMPLEX option in *Mplus*, as implemented and discussed by Asparouhov (2005, 2006), specifying schools as a cluster variable and the robust full-information maximum likelihood estimation. It is important to note that the default estimator for dichotomous items in *Mplus* is WLSMV, but we changed the estimator to be congruent with the following equating process where MLR will be used. Technically speaking, WSLMV analyses tetrachoric correlations that belong to (weighted) least-squares estimation of limited information from the first- and second-order moments, whereas MLR analyses the raw data (full information from all moments)^6^. Reliability was reported for each item (R-squared) and alpha-cfas^7^ were reported for both reduced forms. Item fits were calculated using the procedure described by Yen (1981). This test verifies how much the abilities of the subjects are cited in the characteristic curve suggested by the model, and therefore, rejecting a null hypothesis indicates maladjustment^8^.

Since the parameters from Form A and Form B need to be on the same scale, IRT parameter linking was conducted as the third step. The relationship between item parameters on the two test forms was transformed *via* linking constants (also known as equating coefficients or scaling constants). The linking constants were obtained from the discrimination and difficulty parameters of the anchor items. This step was implemented with the R package equateIRT (Battauz, 2013, 2015; Wiberg, 2018), where different linking methods were used, both relying on item characteristic curves to generate the equating coefficients, namely, the Haebara and Stocking–Lord methods (Haebara, 1980; Stocking and Lord, 1983). The final step in equating transforms the linked scores into a new metric (Bandalos, 2018); therefore, the results of the two equating methods are reported using both IRT observed-score equating (OSE) and IRT true-score equating (TSE) methods. According to Wiberg (2018), the OSE method uses the marginal score distributions, i.e., equipercentile equating is applied to the assumed distributions of the abilities of the examinees which are integrated (summed up) from both forms of the test. On the other hand, the TSE method uses conditional score distributions, and its linking process is associated with the true score obtained in a version of the test to the true score of the other version. Both the methods produce similar results, mainly when the differences between the equated forms are not large (Han et al., 1997).

Lastly, for speech-language pathologists and psychologists, we generated percentiles for the raw scores for each form based on the best fit age-specific reference interval method, as described by Altman (1993), Royston and Sauerbrei (2008), and Royston and Wright (1998). Age-reference intervals are commonly adopted as decision-making tools to determine if an individual is within the normal population interval limits for some measurement (Horn and Pesce, 2005). Classically, age-reference intervals are used in the pediatrics for tracking the child growth across different anthropometric measures (i.e., weight-for-age and weight-for-length/height). It is important to notice that reference-specific age- interval is not correlated with the percentile equating procedure. We used the NCSS version 12 for the reference-specific age-interval method (NCSS 12 Statistical Software, 2018). As both forms will be in the same metric, percentiles will be presented only for Form B, using the OSE method.

## Results

**Figure 1:**
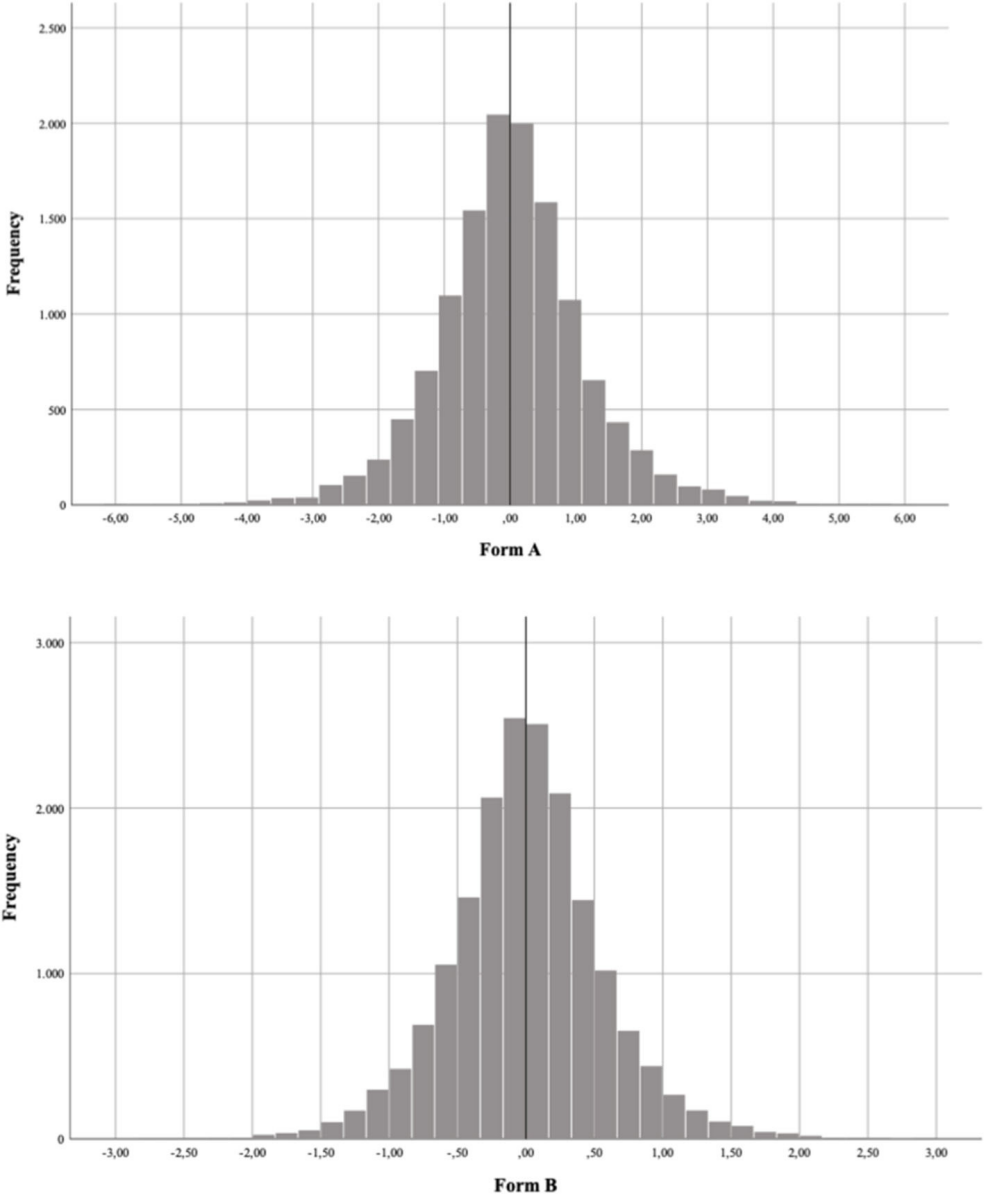
Distribution of the standardized residual (z-scores) for Form A (upper graphic) and Form B (lower graphic).

Figure 1 shows the distribution of the standardized residual (z-scores) separately for Forms A and B, with 12.960 and 17.860 bivariate residuals, respectively. This inspection was important to identify likely violations of local independence. It may be noticed that, in both scenarios, most of the bivariate standardized residuals are around zero: Residuals for Form A ranged from −4.60 to 4.98 (mean = −0.005. SD = 0.591) and residuals for Form B ranged from −4.40 to 4.86 (mean = 0.003; S.D. = 0.578). For Figure 1, the major density of standardized residuals is between −3 and +3 and, therefore, no meaningful deviations were observed, meaning that we have evidence for local independence.

Supplementary Table 1 presents the classification of items and summarizes the statistics per item of Form A and Form B, separately estimated through robust maximum likelihood estimation (only noncommon items). It also presents the discrimination (*a*) and difficulty (*b*) IRT parameters as well as reliability estimates (R-squared) followed by their respective standard errors. The proportion of correct responses for items is presented in the last column. Considering the total of the items, the mean reliability was set to 0.230 for Form A [0.000–0.071] and 0.185 for Form B [0.003–0.442], respectively.

As explained in the Methods section, classification of questions and IRT discrimination/difficulty indices were used to select the items on each Form and for the classification of items [i.e., selecting the items with the highest discrimination with a wide range of difficulty, when possible (−3 to +3), and paired in terms of classification of items]^9^. From these criteria, we selected 33 items specific for each form, which summed up with the 9 anchor items with a total of 42 items by form. The selected items are in bold in Supplementary Table 1 and the last column of Table 2 presents the number of selected items for each text. Item fits are presented in Supplementary Table 2 [χ^2^, df, root mean square error of approximation (RMSEA) associated with χ^2^and *p*-values] The inspection of Supplementary Table 2 shows misfit in five items in Form A (Anchor 4, A42, A52, A55, and A63) and two items in Form B (B9, B93). Item fit was not possible to compute two items (A73 and B95) because the ability level of the sample does not cover the difficulty of those items. We decided to keep these items because removing the misfitting items is relevant when a large proportion of misfits are large or in the case of multidimensionality (Crişan et al., 2017). Supplementary Figure 1 presents the empirical plots for these items. Mean of reliability of the selected items was 0.252 for Form A [0.099– 0.680] and 0.247 for Form B [0.136– 0.606] and it did not differ statistically [*t*_(64)_ = 0.039, *p* = 0.096, *d* = 0.001].

As a next step, we present IRT parameters for the noncommon items together with the anchor items, separated by forms (Supplementary Table 3). This step was carried out using equateIRT for R. The obtained indices did not differ neither for discrimination [*t*_(82)_ = 0.813, *p* = 0.419, *d* = 0.178] nor for difficulty [*t*_(82)_ = 0.870, *p* = 0.387, *d* = 0.265]. Hereafter, we allude to the forms as Form A-R and Form B-R (meaning, they were reduced forms of the original set of items). Alpha-CFAs were similar in both reduced forms [Form A-R: 0.71 (0.67; 0.75); Form B-R: 0.68 (0.64; 0.72)].

From this analysis, the four methods generated different scaling coefficients (Table 4). All methods produced equating coefficients of A > 0.90 and the mean-sigma method produced the highest standard error. The linking was performed putting Form A-R on the scale of Form B-R in all methods. For example, to transform the *a*-parameter for Anchor 1 item from Form A-R (*a* = 1.285143) to the scale of Form B-R using the Stocking–Lord method, we should divide it by 0.91536 (i.e., its equating coefficient A), obtaining the value, 1.403976. The *b*-parameter for Anchor 1 (*b* = 0.835929) from Form A-R is transformed by taking −0.21297 (i.e., equating coefficient B) + 0.91536 ^*^(0.835929) = 0.5522. This procedure, in practice, should be applied to the whole set of items (anchors and non-anchor items).

**Table 4:** Linking coefficients obtained by different methods (Form A–R on Form B–R).

**Method**	**Equating coefficients**			
	**A**	***SE* (A)**	**B**	***SE* (B)**
Haebara	0.93043	0.078377	−0.22625	0.090175
Stocking-Lord	0.91536	0.078744	−0.21297	0.089296

*SE, standard error*.

Supplementary Tables 4 and 5 depict, respectively, the OSE and the TSE-linked scores for Form A-R an Form B-R, based on total scores (OSE) and on theta (TSE). The mean values obtained for Form A-R through OSE were very similar to those obtained through TSE. Using Stocking-Lord method as example, there was an almost perfect correlation between the OSE and the TSE-transformed scores and these measure with the raw score of Form A-R (for all comparison. *r* = 1.0. *p* < 0.001).

**Figure 2:**
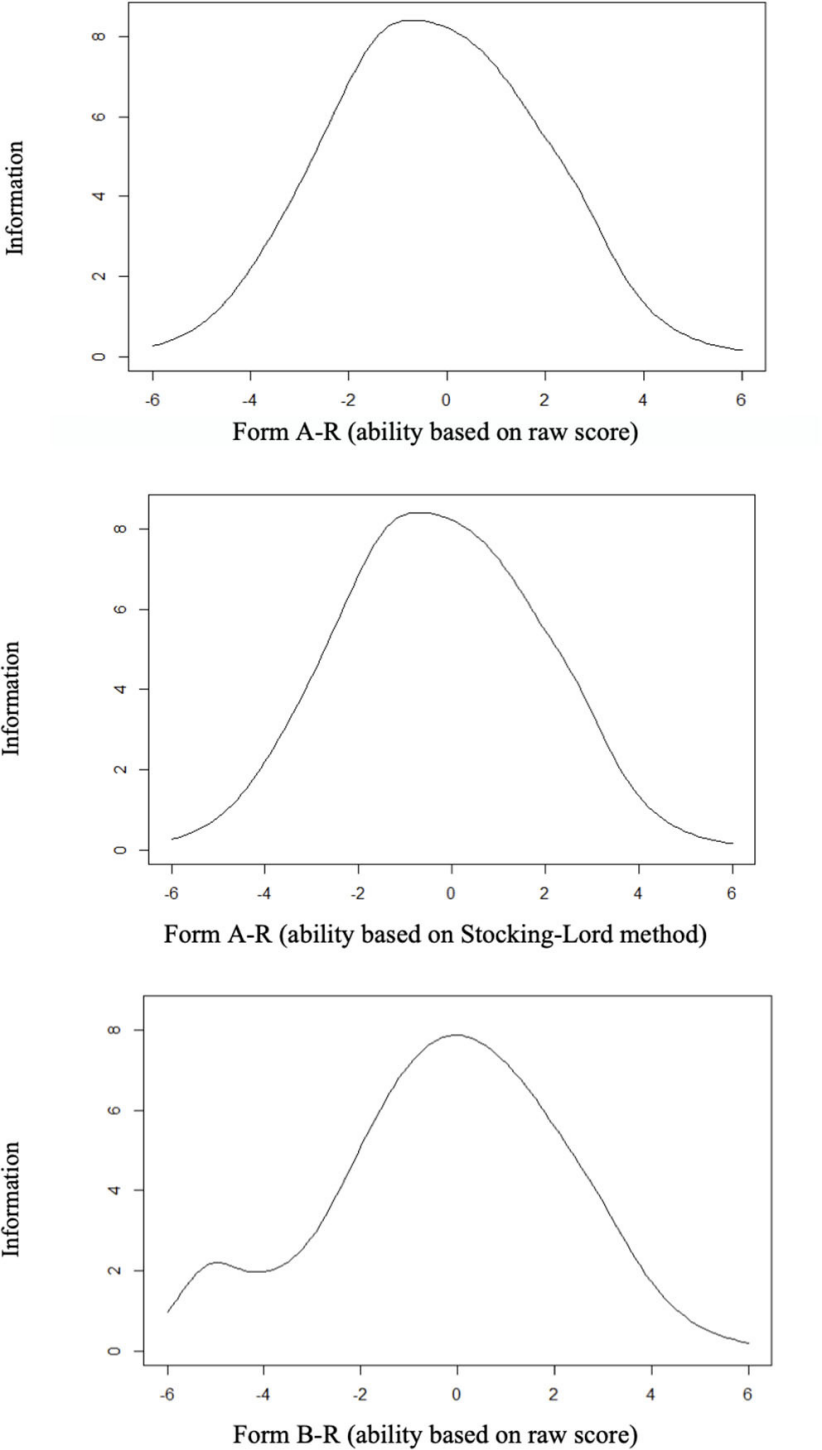
Test information for Form A-R (upper graphics) and Form B-R (lowest graphic).

Figure 2 presents the test information for Form A-R (upper graphics) and Form B-R (lowest graphic). For both forms, the test was informative for a wide range of ability levels, going from −4.00 to 4.00. The peak of information was at theta around −1.0 for Form A-R (Stocking-Lord graphic) and at 0.0 for Form B-R. Greater differences between the information curves seem to be at the lowest bound of the curve.

Reference-specific age-intervals are presented in Table 5 and the percentiles per age were generated using the OSE method. These intervals were obtained based on the highest *R*^2^ correlating age and B-R scores across 44 different models as linear, polynomial (i.e., quadratic and cubic polynomials), a fractional polynomial, and adding an inverse squared term. The *R*^2^ for each fitted model is generated and ranked in terms of its magnitude. The results presented in Table 5 came from the best-fit model. The best model has a quadratic function of growing as the best-fit model with *R*^2^= 0.206. The estimated model for Form B-R scores might be represented by the Equation 1; in case of using decimals, age is not shown in Table 5 (as for e.g., 7.8 years old).


(2) $$\begin{array}{l} B-R\;scores\;=\;47.25+4351.03*\frac{1}{age^{2}}+\;\frac{\log\left(age\right)}{{age}^{2}}*\left(3263.93\right) \end{array}$$

**Table 5:** Reference-specific age-intervals based on total scores on the instrument.

**Age**	**Percentiles**					
	**2.5**	**10**	**25**	**50**	**75**	**90**
7	0	4	7	10	13	16
8	2	5	8	12	16	18
9	4	8	11	15	18	22
10	7	10	14	18	21	24
11	10	13	17	20	23	26

Before using the percentile norms for Form A-R, practitioners should convert the raw score according to one of the four given methods (Supplementary Table 3 or 4). For example, let us consider two children of the same age (8-year-old) who did obtain the score of 16 (child A in Form A-R and Child B in Form B-R). Using Table 5, child B would be achieving the 75th percentile. To compare the performance of both children, we could use Stocking-Lord OSE conversion (Supplementary Table 3) to discover that score of 16 in Form B-R refers to a score of 15.5963 in Form A-R. Therefore, in verbatim terms, child B presented lower achievement than child A. The score of 15.6 is not enough for the examinee to achieve the 75th percentile according to Table 5. Considering the approximation (i.e., values above the 0.50 decimal going up to the next decimal place), both children would achieve similar levels of abilities. Therefore, for practical reasons, such caution would not be necessary, because the test does not present decimals in raw scores and the obtained values from Supplementary Tables 2 and 3 were quite similar.

## Discussion

This study presented the steps for equating two parallel forms of reading comprehension tests to evaluate the abilities of Brazilian Portuguese-speaking children attending the early years of elementary school. The tests (a set of narrative and expository texts, followed by questions) were constructed to cover a wide range of reading comprehension skills, represented by an increasing order of difficulty (i.e., number of words, text complexity, type of texts, and type of questions). Regarding the type of questions, the items differed in the strategies necessary to be evoked for achieving comprehension, i.e., inferences, mental representation of the whole situation, or memorization from information given in the text (Yuill and Oakhill, 1996; Kintsch, 1998; Cain et al., 2004; Paul and Elder, 2012). Following this approach, construct validity was previously demonstrated for the tasks, and the present work aimed to attest empirically its status as parallel forms. For our purposes, we followed the definitions of Kolen (1981) for parallel tests and Bandalos (2018) and Kolen and Brennan (2014) for equating.

We followed the recommendations of Whitley (1983) for constructing a test that simultaneously considered the construct representation and the nomothetic span approaches. In the first case, the texts and questions were developed to access different kinds of inferences that may be used for understanding a passage. In the second case, we encouraged the emergence of the interindividual differences between the children to increase the difficulty of the texts and questions that allowed for catching age differentiation in reading comprehension. Embretson and Gorin (2001) exposed an important role the cognitive psychology would take in the future (where we are, considering the date of the paper) for test construction and test validation. In this study, the theory of the inferential processing guided the construction of the questions and the item selection. We should demonstrate in the future, the role of these inferential processes for construct validation of the task (i.e., comparing potential competing models for this set of items) as well as its external validation (i.e., demonstrating that the nomothetic span principle was, in fact, achieved).

From the recommendations of Kolen and Brennan (2014), the equating process starts with the choice of an appropriate research design. Therefore, a stratified random sampling was performed, and the common-item non-equivalent groups were used as a design. For reducing bias, each half of the sample was randomly assigned to one form of the task and some common items (anchor items) were applied to the whole sample. Some digression here is valuable for explaining our methodological approach. Although the subjects were randomly allocated in our study, the design was considered non-equivalent because no baseline measure was used for testing the *a priori* performance of the participants. Using the stratified random sampling, we intended to reduce the bias and the discrepancy among the groups of subjects. Moreover, we adopted a conservative approach because we used a horizontal instead of vertical equating (Kolen and Brennan, 2014, chapter 9). That is, all the age groups responded to the complete test. As pointed out by a reviewer, using the vertical equating, the younger children could, for example, responded to the easier texts and the older to the medium and hard ones. As mentioned in the Introduction, at the time of data collection, in Brazil, there were scarce literature concerning the test-construction of reading comprehension. We intended providing as many items and texts as possible in the sample, making the results available for the audience.

As the items were embedded in texts, a possible violation of local independence (Embretson and Reise, 2000) could have interfered with score interpretation. For example, using Rasch dichotomous modeling, Moghadam and Pishghadam (2008) showed that local independence violations affected the scores of low- and high-ability students in Cloze tests. Hence, we tested this hypothesis through bivariate Pearson standardized residuals (Haberman, 1973; Agresti, 2019) and employed the Bonferroni's correction for multiple comparisons (i.e., dividing the critical value of 0.05 for the number of items in each form). We found no evidence for the violation of local independence. Note that, even for the traditional cutoff (i.e., |1.96|) used for tests with fewer items (≤ 30), the forms do not seem to present such a violation. As seen in Figure 1, few items surpass |2.0| and even fewer surpass |3.0|.

Having no evidence of local independence violations, we followed the next steps of the analysis proposed on the method. To avoid the anchor length effect, we reduced the original 176 items (81 from Form A and 95 from Form B) to fit 20% of anchor item ratio. Therefore, we reduced both forms to 33 specific items plus 9 common anchor items (called, Form A-R and Form B-R, respectively; R from reduced). Several studies showed that 20% of the rule of thumb is applicable to real and simulated data (Uysal and Kilmen, 2016). For selecting the items, our approach simultaneously considered theoretical and empirical issues. In the first case, we preserved the same number and type of questions per text, to guarantee the conceptual parallelism between the forms. Second, we chose the more discriminative items and sought to achieve heterogeneity in terms of difficulty, which could improve the range of abilities covered by the instrument (Embretson and Reise, 2000; Urbina, 2014). With this approach, we intended to cover from lower to higher levels of reading comprehension skills. In fact, as shown in Figure 2, both forms function equally well for a wide range of theta levels; Therefore, we posit that our aim was achieved. Notably, the score necessary for obtaining the 90th percentiles by the older children of the sample (27 from 45 items; Table 5) indicates that the more difficult items may function for evaluating reading comprehension abilities of even older children^10^. Although, the range of theta obtained for both tasks are satisfactory for our purpose, future research may indicate the applicability of the test for older children.

Consistent with the one-dimensional view of the instrument (Kolen, 1981), separate CFA (Mplus syntax) was used to generate the IRT parameters of difficulty and discrimination for the process of selecting items. After the selection process, we used the equateIRT R package to generate IRT parameters of the reduced forms (Supplementary Table 3). Strikingly, even before equating the linkage, the IRT difficulty and discrimination indices did not differ between the versions. Reliability did not differ significantly between the forms (Kolen, 1981), where almost 71% of the variance in Form A was attributed to true score and 68% was attributed to Form B These results are indicative of the parallelism between forms (Kolen and Brennan, 2014). Subsequent steps involved the estimation of the equating coefficients using two methods (Table 4) and reporting test-equating results based on OSE and TSE methods (Supplementary Tables 4 and 5, respectively). We equated Form A-R onto Form B-R scale. In agreement with the other studies (Ogasawara, 2001; Kilmen and Demirtasli, 2012; Uysal and Kilmen, 2016), Stocking–Lord method presented the lower standard error. Therefore, we demonstrated the score derivation of Form A-R using the latter.

## Limitations, Strengths, and Future Directions

As limitations, we first emphasize that although this study provided some evidence for the interchangeability of the forms, it is necessary to explore the validity for this instrument, such as cross-validation of the results for other samples (e.g., criterion validity for distinguishing good and poor comprehenders, test–retest reliability, or other measures of consistency, such as temporal invariance, and so forth) (American Educational Research Association et al., 2014). Since our conservative approach of not assuming a priori that the groups were equivalent, the representativeness of the common items became relevant, and we had to remove some items to perform the equating. It does not preclude additional analysis to be performed as a part of cross validation studies, using an equivalent random groups approach without the common items, skipping the step of the linking (as gently pointed by a reviewer). This approach seems promising, once the difficult IRT and discrimination index did not differ significantly among the samples even before equating. Sample size limits the analysis to *a posteriori* approach.

Although the final version of the forms preserved comparable comprehension question types, the referenced-age percentiles might be more useful for normative comparison purposes than for the investigation of the cognitive processes involved in the comprehension itself. Therefore, for a complete investigation of the cognitive processes involved in reading comprehension, qualitative guidelines should be given to practitioners. A challenge to this lies in the limited number of questions by the evoked cognitive process, given the pool of items is reduced after item selection. One possibility lies in using some of the questions that were removed but presented an appropriate discrimination index and reliability (e.g., A39 and B45; Supplementary Table 1). Moreover, we infer from Table 5 that a ceiling effect may be present in the task. It signalizes the need for extending the sample for older children (mainly for Texts 6 and 7). Another possibility is for validating other arrangements of the items (e.g., Texts 1–5) for the youngest or for the less skilled. Although feasible, this work is yet to be done. Finally, some steps for equating parallel forms as purposed by von Davier (2013) were not investigated here, such as reliability investigation, symmetry, and invariance for subpopulations. Therefore, future studies should demonstrate these additional steps.

As strengths, we emphasize the sophisticated detailed process description used, which might be used for future research intending to create distinct test forms. We should recognize the careful sampling by stratification, the randomization of Forms A and B to the examinees, the theoretical support for the construction of texts and questions, and the choice of the equating method, which allowed us to offer to the practitioners two interchangeable tools even if a reduced sample size is used. Finally, we reinforce that, once IRT transformations are applied to the item level (Bandalos, 2018), it is possible to create different forms of the instrument, which may be used for different purposes. This is particularly important for both practitioners and researchers in a low-to-middle developing country that does not present yet an instrument with the features described in this study.

In conclusion, the present study fulfilled the steps for demonstrating the equating process of two alternate forms of a reading comprehension test for Brazilian children. Based on the results, we are quite confident that both forms can be used interchangeably, such that the reference-specific age-intervals may be useful for research or clinical/educational purposes. Nevertheless, we recognize that additional steps should be performed as recommended by von Davier (2013). Future research should cross-validate the results for other samples, such as older children or samples with specific learning difficulties, providing further evidence for its diagnostic specificity.

## Data Availability Statement

The raw data supporting the conclusions of this article will be made available by the authors upon formal request, without undue reservation.

## Ethics Statement

The studies involving human participants were reviewed and approved by Ethical Committee of the Federal University of Sao Paulo. Written informed consent to participate in this study was provided by the participants' legal guardian/next of kin.

## Author Contributions

PL conceived the equating design, the research question, and wrote the paper. HC-M performed the sampling process. HC-M and FL analyzed and interpreted the data. DB guided the data analysis and contributed with theoretical and methodological insights. CC and AK participated in data collection, trained the speech language therapists of the study, and reviewed the manuscript. CC, AK, PL, HC-M, and CÁ conceived the instrument. CÁ reviewed the research article. All authors read, revised, and approved the final manuscript.

## Funding

The authors are grateful to Fundação de Amparo à Pesquisa de São Paulo for funding this research (n°. 2011/11369-0).

## Conflict of Interest

The authors declare that the research was conducted in the absence of any commercial or financial relationships that could be construed as a potential conflict of interest.

## Publisher's Note

All claims expressed in this article are solely those of the authors and do not necessarily represent those of their affiliated organizations, or those of the publisher, the editors and the reviewers. Any product that may be evaluated in this article, or claim that may be made by its manufacturer, is not guaranteed or endorsed by the publisher.

## Footnotes

^1^There is no consensus in the literature about the classification of the inferences. Text-connecting inferences are also called as local cohesion inferences and gap-filling are referred as global coherence inferences (Cain and Oakhill, 2014).

^2^Cogo-Moreira, H., Lima, G., Carvalho, C. A. F., Kida, A. B. S., Lúcio, P. S., and Ávila, C. R. B. (2021). Pontos de corte, sensibilidade e especificidade para triagem da fluência leitora em crianças. [Cut-off Scores, Sensitivity, and Specificity for Screening the Reader Fluency in Children].

^3^Here, difficulty should not be interpreted as a statistical measure (such as proportion of correct responses in classical terms or parameter *b* from IRT), but the supposedly increase in difficulty level based on the number of words and the readability of the text.

^4^As the focus of the instrument was to evaluate reading comprehension, we did not specify the way the children should read. In doing this, children who prefer reading silently for understanding the passage (e.g., because she/he is shy) or otherwise who prefer making use of the articulatory help of reading aloud, could be in disadvantage and therefore their score could not represent the actual level of comprehension.

^5^At the time of data collection (2012), we were not aware of the 20% rule of thumb for the anchor items, what would allow that equating and item selection be performed simultaneously. As consequence, first, we removed the items and later we performed the equating.

^6^Using MIRT package would be an alternative for reporting the traditional fit index. Nevertheless, it has some limitations as for example regarding fit indices are only available under complete cases (Chalmers, 2012) and in our cases, missingness was planned by design.

^7^According to Kelley (2020), alpha-cfa should be used when performing a CFA with true score (tau) equivalence and an estimator which is not WLSMV, so the formula is not equivalent to Cronbach's coefficient. This coefficient is also called Guttman-Cronbach alpha and is based on the (1999) work by McDonald.

^8^This statistic fails to find degrees of freedom in very difficult items, so it cannot test the entire length of the curve. Although there are methods with greater power like Orlando and Thiessen's S-χ^2^, we useχ^2^ because it can handle with missing data (Orlando and Thissen, 2000).

^9^For item selection, it was mandatory to preserve the match between the items for both forms. Because of this criterion, it was not always possible to keep the best items in terms of discrimination or reliability. For example, item A6 presented better index than item A7 (discrimination was 0.612 vs. 0.603; and reliability was 0.102 vs. 0.099). Nevertheless, item A7 was selected because it matched with another item, B7. They are of the same classification (GAP), similar in terms of difficulty (A7 = −1.3; B7 = −1.5) and discrimination (B7 = 0.85) and present similar proportion of correct responses (A7 = 68%; B7 = 76%) (Supplementary Table 1).

^10^Another evidence was that fit was not computed for two items (A73; B95) because the ability level of the sample did not cover their difficulty.
