# Supplementary material for: Reading Comprehension Tests for Children: Test Equating and Specific Age-Interval Reports
Source: Front Psychol. 2021 Sep 10;12:662192. doi: 10.3389/fpsyg.2021.662192 (PMC8460877; doi:10.3389/fpsyg.2021.662192)
Supplement: Supplementary file 1 [file Data_Sheet_1.docx]

Supplementary Material

**Table S1.** Discrimination and Difficulty Parameters (IRT) and Proportion of Correct Responses for the Items Pertaining to Forms A and B of the Instrument (Robust Maximum Likelihood Estimation – Mplus Syntax)

| Item | Type | *a* | *a* (SE) | *b* | *b* (SE) | Estimate | E(SE) | Proportion |
| --- | --- | --- | --- | --- | --- | --- | --- | --- |
| A1 | LIT | .552 | .181 | -3.616 | 1.029 | .085 | .051 | .869 |
| A2 | TC | .308 | .108 | -3.576 | 1.466 | .028 | .019 | .746 |
| A3 | TC | .176 | .164 | -12.673 | 11.871 | .009 | .017 | .901 |
| **A4** | **LIT** | **.783** | **.125** | **-.079** | **.181** | **.157** | **.042** | **.514** |
| **A5** | **TC** | **.703** | **.188** | **-2.407** | **.579** | **.130** | **.061** | **.824** |
| A6 | TC | .612 | .252 | -4.137 | 1.563 | .102 | .075 | .915 |
| **A7** | **GAP** | **.603** | **.149** | **-1.317** | **.345** | **.099** | **.044** | **.676** |
| A8 | TC | .564 | .105 | -1.974 | .381 | .088 | .030 | .739 |
| **A9** | **MS** | **.716** | **.110** | **.549** | **.196** | **.135** | **.036** | **.413** |
| A10 | TC | .433 | .084 | -.133 | .241 | .054 | .020 | .514 |
| A11 | TC | .588 | .126 | 1.843 | .446 | .095 | .037 | .268 |
| A12 | TC | .641 | .147 | -1.510 | .356 | .111 | .045 | .709 |
| **A13** | **GAP** | **.947** | **.126** | **-1.496** | **.194** | **.214** | **.045** | **.770** |
| **A14** | **TC** | **.770** | **.146** | **-.641** | **.184** | **.153** | **.049** | **.608** |
| A15 | TC | .523 | .135 | -1.623 | .384 | .077 | .037 | .690 |
| **A16** | **TC** | **1.080** | **.172** | **-2.034** | **.320** | **.262** | **.062** | **.862** |
| A17 | GAP | .736 | .128 | -.740 | .169 | .141 | .042 | .620 |
| **A18** | **LIT** | **.948** | **.192** | **-2.844** | **.438** | **.215** | **.068** | **.913** |
| A19 | MS | .878 | .140 | 1.061 | .241 | .190 | .049 | .310 |
| A20 | MS | .513 | .118 | -.966 | .336 | .074 | .032 | .615 |
| **A21** | **LIT** | **.877** | **.144** | **-.658** | **.150** | **.190** | **.050** | **.622** |
| A22 | TC | .680 | .138 | -.043 | .170 | .123 | .044 | .507 |
| **A23** | **TC** | **1.008** | **.198** | **.505** | **.195** | **.236** | **.071** | **.397** |
| A24 | MS | .006 | .144 | 278.551 | 6240.827 | .000 | .001 | .075 |
| A25 | TC | .489 | .121 | .389 | .245 | .068 | .031 | .455 |
| A26 | GAP | .903 | .138 | 2.730 | .355 | .198 | .049 | .103 |
| **A27** | **TC** | **1.105** | **.206** | **2.927** | **.457** | **.271** | **.074** | **.061** |
| A28 | TC | .402 | .108 | -2.008 | .555 | .047 | .024 | .685 |
| **A29** | **GAP** | **.778** | **.117** | **.598** | **.160** | **.156** | **.039** | **.399** |
| **A30** | **MS** | **.778** | **.169** | **-.708** | **.249** | **.155** | **.057** | **.620** |
| A31 | LIT | .766 | .158 | .945 | .318 | .151 | .053 | .345 |
| **A32** | **GAP** | **1.083** | **.162** | **1.551** | **.312** | **.263** | **.058** | **.202** |
| A33 | GAP | .882 | .137 | .780 | .190 | .191 | .048 | .357 |
| **A34** | **TC** | **1.197** | **.208** | **2.562** | **.333** | **.303** | **.073** | **.075** |
| A35 | LIT | .603 | .161 | .599 | .208 | .100 | .048 | .418 |
| A36 | LIT | .746 | .147 | .858 | .327 | .145 | .049 | .362 |
| **A37** | **TC** | **1.126** | **.187** | **1.276** | **.316** | **.278** | **.067** | **.239** |
| **A38** | **TC** | **1.106** | **.138** | **-.859** | **.149** | **.271** | **.049** | **.683** |
| A39 | TC | 1.375 | .311 | 2.112 | .314 | .365 | .105 | .096 |
| A40 | TC | .629 | .141 | 2.596 | .519 | .107 | .043 | .181 |
| A41 | GAP | .682 | .113 | 1.588 | .217 | .124 | .036 | .272 |
| **A42** | **MS** | **1.021** | **.152** | **.583** | **.204** | **.241** | **.055** | **.380** |
| A43 | GAP | .810 | .123 | -.425 | .200 | .166 | .042 | .575 |
| **A44** | **GAP** | **1.273** | **.202** | **2.318** | **.464** | **.330** | **.070** | **.087** |
| A45 | MS | .748 | .160 | 1.708 | .379 | .145 | .053 | .242 |
| **A46** | **GAP** | **1.462** | **.186** | **.090** | **.180** | **.394** | **.061** | **.477** |
| A47 | GAP | 1.786 | .224 | -.377 | .139 | .492 | .063 | .608 |
| A48 | GAP | 1.372 | .201 | .532 | .193 | .364 | .068 | .369 |
| A49 | LIT | .837 | .183 | -.140 | .180 | .176 | .063 | .526 |
| A50 | LIT | .602 | .094 | -1.909 | .314 | .099 | .028 | .744 |
| **A51** | **TC** | **1.233** | **.153** | **-.563** | **.241** | **.316** | **.054** | **.631** |
| **A52** | **TC** | **2.075** | **.322** | **1.332** | **.185** | **.567** | **.076** | **.155** |
| A53 | TC | 2.852 | .491 | .889 | .136 | .712 | .071 | .225 |
| A54 | GAP | 1.939 | .332 | .899 | .135 | .533 | .085 | .251 |
| **A55** | **TC** | **2.646** | **.439** | **.824** | **.109** | **.680** | **.072** | **.246** |
| A56 | MS | 1.513 | .277 | 1.384 | .213 | .410 | .089 | .181 |
| A57 | MS | 1.696 | .364 | 1.400 | .228 | .466 | .107 | .164 |
| **A58** | **TC** | **1.052** | **.129** | **-.118** | **.160** | **.252** | **.046** | **.526** |
| A59 | GAP | .844 | .141 | -.719 | .141 | .178 | .049 | .629 |
| A60 | GAP | 1.176 | .175 | .435 | .177 | .296 | .062 | .401 |
| A61 | TC | .463 | .116 | -.794 | .454 | .061 | .029 | .587 |
| **A62** | **TC** | **1.324** | **.244** | **.503** | **.193** | **.348** | **.084** | **.378** |
| **A63** | **TC** | **1.109** | **.184** | **.397** | **.131** | **.272** | **.066** | **.413** |
| A64 | GAP | 1.635 | .230 | 1.918 | .198 | .448 | .070 | .096 |
| A65 | GAP | 1.819 | .163 | 1.289 | .150 | .501 | .045 | .176 |
| **A66** | **TC** | **.943** | **.122** | **2.138** | **.337** | **.213** | **.043** | **.150** |
| A67 | TC | .635 | .132 | .893 | .331 | .109 | .041 | .373 |
| A68 | GAP | 1.179 | .216 | .605 | .117 | .297 | .077 | .364 |
| A69 | GAP | 1.279 | .207 | 1.005 | .199 | .332 | .072 | .272 |
| A70 | MS | .846 | .151 | .429 | .150 | .179 | .052 | .423 |
| A71 | MS | 1.467 | .231 | 2.312 | .181 | .395 | .075 | .070 |
| **A72** | **LIT** | **.849** | **.127** | **-.462** | **.167** | **.180** | **.044** | **.585** |
| **A73** | **TC** | **1.452** | **.275** | **2.850** | **.411** | **.391** | **.090** | **.038** |
| A74 | TC | .127 | .394 | 25.661 | 79.964 | .005 | .030 | .038 |
| **A75** | **TC** | **1.334** | **.145** | **2.448** | **.223** | **.351** | **.050** | **.070** |
| **A76** | **GAP** | **1.182** | **.193** | **2.913** | **.421** | **.298** | **.068** | **.054** |
| **A77** | **TC** | **.917** | **.172** | **2.034** | **.304** | **.204** | **.061** | **.167** |
| A78 | GAP | 1.074 | .255 | 3.072 | .587 | .260 | .091 | .056 |
| A79 | MS | .751 | .131 | 1.555 | .292 | .146 | .044 | .261 |
| **A80** | **GAP** | **.995** | **.217** | **1.257** | **.218** | **.231** | **.077** | **.261** |
| A81 | MS | 1.494 | .280 | 2.347 | .271 | .404 | .090 | .066 |
| B1 | TC | .277 | .152 | -6.177 | 3.246 | .023 | .024 | .843 |
| B2 | TC | .582 | .127 | -3.000 | .563 | .093 | .037 | .837 |
| B3 | LIT | .653 | .181 | -3.354 | .819 | .115 | .056 | .884 |
| B4 | TC | .425 | .225 | -7.271 | 3.578 | .052 | .052 | .953 |
| B5 | GAP | .581 | .163 | -3.222 | .922 | .093 | .047 | .853 |
| B6 | TC | .099 | .268 | -2.899 | 57.146 | .003 | .016 | .887 |
| **B7** | **GAP** | **.851** | **.174** | **-1.583** | **.265** | **.180** | **.060** | **.765** |
| B8 | TC | 1.173 | .249 | -1.470 | .298 | .295 | .088 | .799 |
| **B9** | **LIT** | **.742** | **.144** | **-2.214** | **.434** | **.143** | **.048** | **.815** |
| B10 | TC | 1.245 | .302 | -1.208 | .324 | .320 | .105 | .765 |
| **B11** | **TC** | **1.376** | **.230** | **-.384** | **.209** | **.365** | **.077** | **.602** |
| B12 | TC | 1.098 | .228 | -2.144 | .391 | .268 | .081 | .875 |
| B13 | TC | .915 | .182 | .529 | .201 | .203 | .064 | .401 |
| **B14** | **MS** | **1.072** | **.206** | **-1.675** | **.386** | **.259** | **.074** | **.815** |
| B15 | LIT | .339 | .128 | .518 | .386 | .034 | .025 | .458 |
| B16 | GAP | .933 | .169 | -1.307 | .185 | .209 | .060 | .740 |
| **B17** | **LIT** | **1.041** | **.263** | **-2.289** | **.426** | **.248** | **.094** | **.881** |
| B18 | TC | .634 | .161 | -2.293 | .520 | .109 | .049 | .793 |
| B19 | GAP | .939 | .211 | -.692 | .304 | .211 | .075 | .636 |
| B20 | LIT | .620 | .374 | -5.835 | 3.252 | .105 | .113 | .969 |
| **B21** | **TC** | **.999** | **.172** | **-.878** | **.279** | **.233** | **.062** | **.677** |
| B22 | GAP | .783 | .137 | .480 | .200 | .157 | .046 | .420 |
| B23 | GAP | .637 | .162 | -.767 | .193 | .110 | .050 | .611 |
| B24 | GAP | .798 | .147 | -.748 | .248 | .162 | .050 | .630 |
| **B25** | **GAP** | **1.029** | **.214** | **.100** | **.168** | **.243** | **.077** | **.483** |
| B26 | TC | .674 | .179 | -.861 | .294 | .121 | .057 | .630 |
| **B27** | **TC** | **1.193** | **.308** | **-2.586** | **.611** | **.302** | **.109** | **.925** |
| B28 | MS | 1.548 | .246 | -.327 | .178 | .421 | .077 | .590 |
| B29 | LIT | .689 | .202 | -3.801 | 1.051 | .126 | .065 | .918 |
| B30 | TC | .951 | .154 | -.038 | .164 | .216 | .055 | .511 |
| B31 | LIT | .780 | .183 | -1.720 | .400 | .156 | .062 | .768 |
| **B32** | **GAP** | **.940** | **.156** | **.214** | **.147** | **.212** | **.056** | **.461** |
| B33 | GAP | .634 | .163 | .970 | .273 | .109 | .050 | .364 |
| **B34** | **TC** | **1.387** | **.178** | **-1.630** | **.290** | **.369** | **.060** | **.846** |
| **B35** | **LIT** | **1.142** | **.222** | **-1.150** | **.248** | **.284** | **.079** | **.743** |
| B36 | GAP | .922 | .201 | .281 | .200 | .205 | .071 | .448 |
| B37 | GAP | .333 | .131 | 5.316 | 1.927 | .033 | .025 | .150 |
| **B38** | **TC** | **.932** | **.202** | **.344** | **.159** | **.209** | **.072** | **.436** |
| B39 | MS | .634 | .154 | .721 | .265 | .109 | .047 | .398 |
| B40 | TC | .919 | .247 | -.432 | .290 | .204 | .087 | .586 |
| **B41** | **MS** | **.914** | **.177** | **.202** | **.185** | **.203** | **.063** | **.464** |
| B42 | TC | .712 | .234 | 3.248 | 1.179 | .133 | .076 | .107 |
| **B43** | **TC** | **1.167** | **.179** | **1.240** | **.149** | **.293** | **.064** | **.241** |
| B44 | TC | .646 | .119 | -.713 | .243 | .112 | .037 | .605 |
| B45 | LIT | 1.565 | .181 | .814 | .159 | .427 | .056 | .295 |
| B46 | TC | .887 | .253 | -.705 | .336 | .193 | .089 | .633 |
| B47 | TC | .815 | .158 | -1.451 | .408 | .168 | .054 | .740 |
| B48 | TC | .675 | .150 | 1.238 | .314 | .122 | .048 | .320 |
| B49 | TC | .222 | .136 | -5.286 | 3.408 | .015 | .018 | .762 |
| **B50** | **TC** | **1.040** | **.150** | **1.031** | **.209** | **.247** | **.054** | **.295** |
| B51 | GAP | .811 | .195 | .522 | .238 | .167 | .067 | .411 |
| **B52** | **MS** | **1.498** | **.288** | **1.181** | **.191** | **.406** | **.093** | **.219** |
| B53 | TC | .461 | .162 | 4.785 | 1.556 | .061 | .040 | .107 |
| **B54** | **GAP** | **1.615** | **.459** | **2.683** | **.518** | **.442** | **.140** | **.034** |
| **B55** | **TC** | **.983** | **.170** | **1.623** | **.276** | **.227** | **.061** | **.207** |
| **B56** | **GAP** | **.809** | **.264** | **.182** | **.214** | **.166** | **.090** | **.470** |
| B57 | MS | .850 | .313 | 3.397 | 1.036 | .180 | .109 | .069 |
| **B58** | **TC** | **1.126** | **.206** | **-.759** | **.270** | **.278** | **.074** | **.668** |
| B59 | LIT | .945 | .215 | 1.047 | .228 | .213 | .076 | .304 |
| **B60** | **GAP** | **.930** | **.221** | **-.428** | **.298** | **.208** | **.078** | **.586** |
| B61 | TC | .609 | .103 | -.727 | .290 | .101 | .031 | .602 |
| **B62** | **TC** | **.876** | **.227** | **1.428** | **.226** | **.189** | **.079** | **.254** |
| B63 | TC | .559 | .147 | 2.189 | .596 | .087 | .042 | .241 |
| B64 | TC | .477 | .160 | -.007 | .383 | .065 | .041 | .502 |
| B65 | TC | .641 | .177 | 1.414 | .340 | .111 | .054 | .304 |
| **B66** | **TC** | **.837** | **.147** | **.836** | **.240** | **.176** | **.051** | **.354** |
| B67 | GAP | .465 | .147 | .276 | .245 | .062 | .037 | .470 |
| B68 | GAP | .536 | .147 | -.255 | .298 | .080 | .040 | .533 |
| B69 | TC | .258 | .122 | 5.356 | 2.496 | .020 | .018 | .204 |
| B70 | TC | .211 | .124 | 4.706 | 2.861 | .013 | .015 | .273 |
| B71 | LIT | .988 | .204 | -.503 | .250 | .229 | .073 | .605 |
| B72 | TC | .625 | .164 | 1.368 | .355 | .106 | .050 | .313 |
| B73 | TC | .212 | .121 | 4.677 | 2.755 | .013 | .015 | .273 |
| B74 | TC | .446 | .146 | -.215 | .313 | .057 | .035 | .524 |
| **B75** | **TC** | **.863** | **.213** | **1.488** | **.332** | **.185** | **.074** | **.248** |
| B76 | TC | .563 | .205 | -1.294 | .522 | .088 | .058 | .665 |
| B77 | TC | .562 | .233 | 3.362 | 1.124 | .087 | .066 | .144 |
| B78 | TC | .695 | .194 | -.119 | .250 | .128 | .062 | .520 |
| B79 | LIT | 1.423 | .253 | .732 | .173 | .381 | .084 | .323 |
| **B80** | **TC** | **1.126** | **.226** | **-.001** | **.194** | **.278** | **.081** | **.505** |
| B81 | TC | .945 | .229 | -.055 | .212 | .213 | .081 | .514 |
| B82 | TC | .948 | .218 | -1.060 | .317 | .214 | .077 | .702 |
| **B83** | **TC** | **1.236** | **.231** | **.387** | **.158** | **.317** | **.081** | **.414** |
| **B84** | **TC** | **.921** | **.213** | **.678** | **.211** | **.205** | **.075** | **.373** |
| B85 | TC | .629 | .200 | 3.128 | .964 | .107 | .061 | .138 |
| **B86** | **LIT** | **.720** | **.160** | **2.533** | **.570** | **.136** | **.052** | **.160** |
| B87 | TC | .984 | .227 | 1.600 | .323 | .227 | .081 | .210 |
| B88 | TC | .974 | .178 | 2.256 | .390 | .224 | .063 | .132 |
| B89 | TC | 1.203 | .290 | 2.025 | .338 | .305 | .102 | .122 |
| **B90** | **TC** | **1.226** | **.257** | **1.103** | **.170** | **.314** | **.090** | **.260** |
| **B91** | **TC** | **1.126** | **.218** | **1.095** | **.158** | **.278** | **.078** | **.273** |
| **B92** | **TC** | **1.587** | **.297** | **.287** | **.154** | **.434** | **.092** | **.429** |
| **B93** | **GAP** | **.779** | **.218** | **2.311** | **.662** | **.156** | **.074** | **.166** |
| B94 | TC | 1.305 | .205 | 1.556 | .162 | .341 | .071 | .172 |
| **B95** | **GAP** | **2.251** | **.791** | **2.262** | **.384** | **.606** | **.168** | **.034** |

Form A: A1-A9 (Text 1); A10-A20 (Text 2); A21-A30 (Text 3); A31-A45 (Text 4); A46-A57 (Text 5); A58-A71 (Text 6); A72-A81 (Text 7); Form B: B1-B14 (Text 1); B15-B28 (Text 2); B29-B41 (Text 3); B42-B57 (Text 4); B58-B70 (Text 5); B71-B84 (Text 6); B85-B95 (Text 7). Selected items are in bold; *a* = discrimination; *b* = difficult; SE = standard error. E(SE) = standard error of estimate. Type = questions’ classification: LIT = literal; TC = text connection; GAP = gap-filling; SM = situational model.

**Table S2.** Fit Index for the Selected Items.

| Item | *χ^2^* | *df* | *RMSEA(χ^2^)* | *p.χ^2^* | Item | X2 | *df* | *RMSEA(χ^2^)* | *p.χ^2^* |
| --- | --- | --- | --- | --- | --- | --- | --- | --- | --- |
| Anchor1 | 3.592 | 8 | 0.000 | 0.892 | Anchor1 | 8.516 | 7 | 0.026 | 0.289 |
| Anchor2 | 4.629 | 4 | 0.019 | 0.328 | Anchor2 | 1.254 | 2 | 0.000 | 0.534 |
| Anchor3 | 4.117 | 8 | 0.000 | 0.846 | Anchor3 | 7.268 | 8 | 0.000 | 0.508 |
| Anchor4 | 15.989 | 8 | 0.048 | **0.043** | Anchor4 | 3.702 | 8 | 0.000 | 0.883 |
| Anchor5 | 15.060 | 8 | 0.045 | 0.058 | Anchor5 | 7.647 | 8 | 0.000 | 0.469 |
| Anchor6 | 7.705 | 7 | 0.015 | 0.359 | Anchor6 | 7.718 | 6 | 0.030 | 0.260 |
| Anchor7 | 8.659 | 8 | 0.014 | 0.372 | Anchor7 | 5.933 | 8 | 0.000 | 0.655 |
| Anchor8 | 5.661 | 6 | 0.000 | 0.462 | Anchor8 | 6.458 | 5 | 0.030 | 0.264 |
| Anchor9 | 7.432 | 8 | 0.000 | 0.491 | Anchor9 | 2.721 | 8 | 0.000 | 0.951 |
| A4 | 12.391 | 8 | 0.036 | 0.135 | B7 | 7.416 | 8 | 0.000 | 0.492 |
| A5 | 4.678 | 7 | 0.000 | 0.699 | B9 | 15.603 | 8 | 0.054 | **0.048** |
| A7 | 2.878 | 8 | 0.000 | 0.942 | B11 | 7.889 | 8 | 0.000 | 0.444 |
| A9 | 5.335 | 8 | 0.000 | 0.721 | B14 | 3.029 | 6 | 0.000 | 0.805 |
| A13 | 10.761 | 7 | 0.035 | 0.149 | B17 | 6.231 | 4 | 0.042 | 0.183 |
| A14 | 7.604 | 8 | 0.000 | 0.473 | B21 | 7.416 | 8 | 0.000 | 0.493 |
| A16 | 9.559 | 6 | 0.037 | 0.145 | B25 | 6.999 | 8 | 0.000 | 0.537 |
| A18 | 2.620 | 4 | 0.000 | 0.623 | B27 | 2.857 | 1 | 0.076 | 0.091 |
| A21 | 10.925 | 8 | 0.029 | 0.206 | B32 | 10.369 | 8 | 0.030 | 0.240 |
| A23 | 3.185 | 8 | 0.000 | 0.922 | B34 | 2.912 | 4 | 0.000 | 0.573 |
| A27 | 2.529 | 3 | 0.000 | 0.470 | B35 | 12.183 | 7 | 0.048 | 0.095 |
| A29 | 10.300 | 8 | 0.026 | 0.245 | B38 | 5.997 | 8 | 0.000 | 0.648 |
| A30 | 7.096 | 8 | 0.000 | 0.526 | B41 | 8.837 | 8 | 0.018 | 0.356 |
| A32 | 6.148 | 7 | 0.000 | 0.523 | B43 | 5.207 | 6 | 0.000 | 0.518 |
| A34 | 4.959 | 3 | 0.039 | 0.175 | B50 | 5.459 | 7 | 0.000 | 0.604 |
| A37 | 11.263 | 7 | 0.038 | 0.128 | B52 | 10.304 | 6 | 0.047 | 0.112 |
| A38 | 8.083 | 8 | 0.005 | 0.425 | B55 | 7.479 | 7 | 0.015 | 0.381 |
| A42 | 19.495 | 8 | 0.058 | **0.012** | B56 | 8.013 | 8 | 0.002 | 0.432 |
| A44 | 0.969 | 3 | 0.000 | 0.809 | B58 | 6.961 | 7 | 0.000 | 0.433 |
| A46 | 4.192 | 8 | 0.000 | 0.839 | B60 | 6.475 | 8 | 0.000 | 0.594 |
| A51 | 10.465 | 8 | 0.027 | 0.234 | B62 | 2.931 | 7 | 0.000 | 0.891 |
| A52 | 12.301 | 5 | 0.058 | **0.031** | B66 | 9.957 | 8 | 0.028 | 0.268 |
| A55 | 13.905 | 6 | 0.055 | **0.031** | B75 | 7.912 | 8 | 0.000 | 0.442 |
| A58 | 4.415 | 8 | 0.000 | 0.818 | B80 | 7.852 | 8 | 0.000 | 0.448 |
| A62 | 12.081 | 8 | 0.034 | 0.148 | B83 | 11.373 | 8 | 0.036 | 0.181 |
| A63 | 19.124 | 8 | 0.057 | **0.014** | B84 | 8.931 | 8 | 0.019 | 0.348 |
| A66 | 13.725 | 7 | 0.047 | 0.056 | B86 | 3.930 | 7 | 0.000 | 0.788 |
| A72 | 6.727 | 8 | 0.000 | 0.566 | B89 | 8.677 | 4 | 0.060 | 0.070 |
| A73 | 1.169 | 0 | NA | **NA** | B90 | 8.116 | 7 | 0.022 | 0.323 |
| A75 | 2.080 | 3 | 0.000 | 0.556 | B91 | 3.274 | 7 | 0.000 | 0.859 |
| A76 | 5.770 | 2 | 0.066 | 0.056 | B92 | 7.630 | 7 | 0.017 | 0.366 |
| A77 | 5.221 | 7 | 0.000 | 0.633 | B93 | 13.792 | 6 | 0.064 | **0.032** |
| A80 | 10.300 | 8 | 0.026 | 0.245 | B95 | 2.320 | 0 | **NA** | **NA** |

*Note*. Items with misfit identified or not computed are in bold.

**Table S3.** IRT Parameters (One-Dimensional Model) for the Non-Common Items (Forms A and B) and Anchor Items

| Form A-R | | | Form B-R | | |
| --- | --- | --- | --- | --- | --- |
| Item | *b* | *a* | Item | *b* | *a* |
| Anchor1 | 0.835929 | 1.285143 | Anchor1 | 1.173508 | 1.289819 |
| Anchor2 | 2.66795 | 1.810883 | Anchor2 | 2.965689 | 1.685748 |
| Anchor3 | -0.31471 | 0.92365 | Anchor3 | 0.21072 | 0.790233 |
| Anchor4 | 0.303852 | 0.635694 | Anchor4 | 0.314999 | 0.76942 |
| Anchor5 | -0.13014 | 0.733468 | Anchor5 | -0.24372 | 0.878501 |
| Anchor6 | 1.271858 | 1.462151 | Anchor6 | 1.527632 | 1.673012 |
| Anchor7 | -0.01501 | 1.025545 | Anchor7 | 0.285143 | 1.405511 |
| Anchor8 | -2.00348 | 1.27289 | Anchor8 | -1.87349 | 1.307599 |
| Anchor9 | -0.5364 | 0.995811 | Anchor9 | -0.11157 | 1.198234 |
| A4 (LIT) | 0.066866 | 0.803201 | B7 (GAP) | 1.312824 | 0.787645 |
| A5 (TC) | 1.731969 | 0.780383 | B9 (LIT) | 1.630111 | 0.693496 |
| A7 (GAP) | 0.778744 | 0.590024 | B11 (TC) | 0.526517 | 1.362858 |
| A9 (MS) | -0.4022 | 0.766022 | B14 (MS) | 1.80112 | 1.115789 |
| A13 (GAP) | 1.479803 | 1.07382 | B17 (LIT) | 2.368302 | 1.013465 |
| A14 (TC) | 0.511333 | 0.80471 | B21 (TC) | 0.897297 | 1.105073 |
| A16 (TC) | 2.277313 | 1.194419 | B25 (GAP) | -0.10234 | 0.985529 |
| A18 (LIT) | 2.833381 | 1.127314 | B27 (TC) | 3.160506 | 1.278128 |
| A21 (LIT) | 0.59453 | 0.900127 | B32 (GAP) | -0.204 | 0.938869 |
| A23 (TC) | -0.50107 | 1.021775 | B34 (TC) | 2.370728 | 1.535627 |
| A27 (TC) | -3.05016 | 0.920746 | B35 (LIT) | 1.292764 | 1.059369 |
| A29 (GAP) | -0.45881 | 0.783401 | B38 (TC) | -0.31996 | 0.906189 |
| A30 (MS) | 0.546107 | 0.73837 | B41 (MS) | -0.19165 | 0.970608 |
| A32 (GAP) | -1.70278 | 1.136883 | B43 (TC) | -1.49623 | 1.264851 |
| A34 (TC) | -3.03627 | 1.158379 | B50 (TC) | -1.06199 | 1.001392 |
| A37 (TC) | -1.4376 | 1.172155 | B52 (MS) | -1.74216 | 1.440398 |
| A38 (TC) | 0.958857 | 1.093239 | B54 (GAP) | -4.31457 | 1.589796 |
| A42 (MS) | -0.59268 | 1.072542 | B55 (TC) | -1.53653 | 0.841229 |
| A44 (GAP) | -2.92803 | 1.245318 | B56 (GAP) | -0.15117 | 0.818616 |
| A46 (GAP) | -0.11568 | 1.28504 | B58 (TC) | 0.886073 | 1.272997 |
| A51 (TC) | 0.71614 | 1.266427 | B60 (GAP) | 0.402949 | 1.025937 |
| A52 (TC) | -2.29955 | 1.471113 | B62 (TC) | -1.25138 | 0.868902 |
| A55 (TC) | -1.7003 | 1.813747 | B66 (TC) | -0.70515 | 0.848683 |
| A58 (TC) | 0.115238 | 0.955203 | B75 (TC) | -1.25891 | 0.784209 |
| A62 (TC) | -0.65521 | 1.34996 | B80 (TC) | 0.000847 | 1.076744 |
| A63 (TC) | -0.43927 | 1.190534 | B83 (TC) | -0.47174 | 1.197186 |
| A66 (TC) | -1.99881 | 0.947455 | B84 (TC) | -0.62624 | 0.918617 |
| A72 (LIT) | 0.414488 | 0.919326 | B86 (LIT) | -1.77529 | 0.591099 |
| A73 (TC) | -4.10163 | 1.408187 | B90 (TC) | -1.32627 | 1.15873 |
| A75 (TC) | -3.07291 | 1.108456 | B91 (TC) | -1.28287 | 1.243492 |
| A76 (GAP) | -3.28339 | 0.988486 | B92 (TC) | -0.43165 | 1.570617 |
| A77 (TC) | -1.86646 | 0.9193 | B93 (GAP) | -1.85594 | 0.891635 |
| A80 (GAP) | -1.25486 | 1.051198 | B95 (GAP) | -5.20511 | 2.327593 |

*Note*. *a* = discrimination; *b* = difficult; R = reduced (42 items per form); SE = standard error. In parenthesis. questions’ classification: LIT = literal; TC = text connection; GAP = gap-filling; SM = situational model. Text 1 (A4-A9; B7-B14); Text 2 (A13-A18; B17-B24); Text 3 (A21-A30; B32-B41); Text 4 (A32-A44; B43-B58); Text 5 (A46-A55; B58-66); Text 6 (A58-A66; B75-B84); Text 7 (A72-A80; B86-B95). Anchor items’ classification: TC = 1, 2, 4, 6, 7; GAP = 3, 8; LIT = 5; MS = 9.

**Table S4.** Observed Score Equating (OSE) Based on Two Different Methods

| Form B-R (Raw score) | Haebara | | Stocking-Lord | |
| --- | --- | --- | --- | --- |
|  | A on B | SE | A on B | SE |
| 0 | -0.0390 | 0.3081 | -0.1114 | 0.2593 |
| 1 | 0.9435 | 0.3871 | 0.8444 | 0.3405 |
| 2 | 1.9250 | 0.4374 | 1.8031 | 0.3982 |
| 3 | 2.9063 | 0.4674 | 2.7653 | 0.4376 |
| 4 | 3.8884 | 0.4831 | 3.7320 | 0.4629 |
| 5 | 4.8722 | 0.4886 | 4.7039 | 0.4770 |
| 6 | 5.8578 | 0.4864 | 5.6811 | 0.4815 |
| 7 | 6.8451 | 0.4789 | 6.6634 | 0.4786 |
| 8 | 7.8336 | 0.4681 | 7.6496 | 0.4709 |
| 9 | 8.8223 | 0.4554 | 8.6387 | 0.4594 |
| 10 | 9.8105 | 0.4408 | 9.6301 | 0.4440 |
| 11 | 10.7979 | 0.4258 | 10.6230 | 0.4269 |
| 12 | 11.7845 | 0.4122 | 11.6163 | 0.4109 |
| 13 | 12.7703 | 0.4008 | 12.6097 | 0.3969 |
| 14 | 13.7553 | 0.3910 | 13.6037 | 0.3837 |
| 15 | 14.7401 | 0.3831 | 14.5993 | 0.3716 |
| 16 | 15.7255 | 0.3791 | 15.5963 | 0.3634 |
| 17 | 16.7120 | 0.3801 | 16.5944 | 0.3614 |
| 18 | 17.7003 | 0.3860 | 17.5945 | 0.3649 |
| 19 | 18.6916 | 0.3963 | 18.5987 | 0.3731 |
| 20 | 19.6869 | 0.4114 | 19.6083 | 0.3870 |
| 21 | 20.6858 | 0.4320 | 20.6221 | 0.4078 |
| 22 | 21.6878 | 0.4571 | 21.6385 | 0.4343 |
| 23 | 22.6935 | 0.4854 | 22.6584 | 0.4646 |
| 24 | 23.7045 | 0.5164 | 23.6843 | 0.4983 |
| 25 | 24.7214 | 0.5504 | 24.7170 | 0.5359 |
| 26 | 25.7417 | 0.5861 | 25.7532 | 0.5757 |
| 27 | 26.7628 | 0.6216 | 26.7889 | 0.6149 |
| 28 | 27.7839 | 0.6559 | 27.8239 | 0.6525 |
| 29 | 28.8063 | 0.6896 | 28.8598 | 0.6892 |
| 30 | 29.8296 | 0.7225 | 29.8963 | 0.7250 |
| 31 | 30.8509 | 0.7539 | 30.9297 | 0.7580 |
| 32 | 31.8679 | 0.7834 | 31.9572 | 0.7872 |
| 33 | 32.8802 | 0.8116 | 32.9787 | 0.8138 |
| 34 | 33.8886 | 0.8390 | 33.9951 | 0.8383 |
| 35 | 34.8933 | 0.8648 | 35.0064 | 0.8590 |
| 36 | 35.8947 | 0.8872 | 36.0123 | 0.8739 |
| 37 | 36.8949 | 0.9030 | 37.0149 | 0.8800 |
| 38 | 37.8979 | 0.9055 | 38.0175 | 0.8708 |
| 39 | 38.9084 | 0.8842 | 39.0236 | 0.8366 |
| 40 | 39.9295 | 0.8257 | 40.0350 | 0.7658 |
| 41 | 40.9606 | 0.7153 | 41.0497 | 0.6470 |
| 42 | 41.9925 | 0.5447 | 42.0580 | 0.4762 |

*Note*. A on B = expected raw score in Form A-R based on the respective raw score on Form B-R; SE = standard error.

**Table S5.** True Score Equating (TSE) Based on Two Different Methods

| Theta | Form B-R (Raw score) | Haebara | | Stocking-Lord | |
| --- | --- | --- | --- | --- | --- |
|  |  | A on B | SE | A on B | SE |
| -4.2825 | 1 | 0.9953 | 0.3179 | 0.9290 | 0.299763 |
| -3.4791 | 2 | 1.9771 | 0.4533 | 1.8679 | 0.438211 |
| -2.9997 | 3 | 2.9515 | 0.5206 | 2.8108 | 0.513286 |
| -2.6491 | 4 | 3.9232 | 0.5513 | 3.7589 | 0.552761 |
| -2.3676 | 5 | 4.8963 | 0.5614 | 4.7144 | 0.570911 |
| -2.1286 | 6 | 5.8731 | 0.5587 | 5.6789 | 0.574595 |
| -1.9181 | 7 | 6.8543 | 0.5477 | 6.6525 | 0.567477 |
| -1.7277 | 8 | 7.8390 | 0.5312 | 7.6340 | 0.552283 |
| -1.5521 | 9 | 8.8255 | 0.5116 | 8.6211 | 0.531539 |
| -1.3878 | 10 | 9.8122 | 0.4906 | 9.6117 | 0.507545 |
| -1.2322 | 11 | 10.7981 | 0.4694 | 10.6040 | 0.482198 |
| -1.0834 | 12 | 11.7823 | 0.4491 | 11.5968 | 0.456965 |
| -0.9400 | 13 | 12.7651 | 0.4304 | 12.5897 | 0.433006 |
| -0.8011 | 14 | 13.7468 | 0.4141 | 13.5827 | 0.411343 |
| -0.6656 | 15 | 14.7282 | 0.4011 | 14.5766 | 0.392992 |
| -0.5328 | 16 | 15.7102 | 0.3922 | 15.5719 | 0.379027 |
| -0.4021 | 17 | 16.6939 | 0.3884 | 16.5696 | 0.370563 |
| -0.2730 | 18 | 17.6804 | 0.3904 | 17.5708 | 0.368646 |
| -0.1448 | 19 | 18.6707 | 0.3987 | 18.5763 | 0.374084 |
| -0.0173 | 20 | 19.6659 | 0.4134 | 19.5872 | 0.387271 |
| 0.1102 | 21 | 20.6667 | 0.4343 | 20.6041 | 0.408091 |
| 0.2380 | 22 | 21.6736 | 0.4608 | 21.6275 | 0.435941 |
| 0.3667 | 23 | 22.6870 | 0.4920 | 22.6576 | 0.469835 |
| 0.4967 | 24 | 23.7065 | 0.5270 | 23.6940 | 0.50854 |
| 0.6284 | 25 | 24.7316 | 0.5647 | 24.7361 | 0.5507 |
| 0.7625 | 26 | 25.7612 | 0.6039 | 25.7825 | 0.594942 |
| 0.8996 | 27 | 26.7940 | 0.6435 | 26.8315 | 0.639986 |
| 1.0401 | 28 | 27.8281 | 0.6828 | 27.8814 | 0.684724 |
| 1.1850 | 29 | 28.8618 | 0.7210 | 28.9301 | 0.728283 |
| 1.3348 | 30 | 29.8930 | 0.7579 | 29.9752 | 0.770047 |

*Note*. A on B = expected raw score in Form A-R based on the respective raw score on Form B-R; SE = standard error.
