# Supplementary figures and images for: Reading Comprehension Tests for Children: Test Equating and Specific Age-Interval Reports
Source: Front Psychol. 2021 Sep 10;12:662192. doi: 10.3389/fpsyg.2021.662192 (PMC8460877; doi:10.3389/fpsyg.2021.662192)

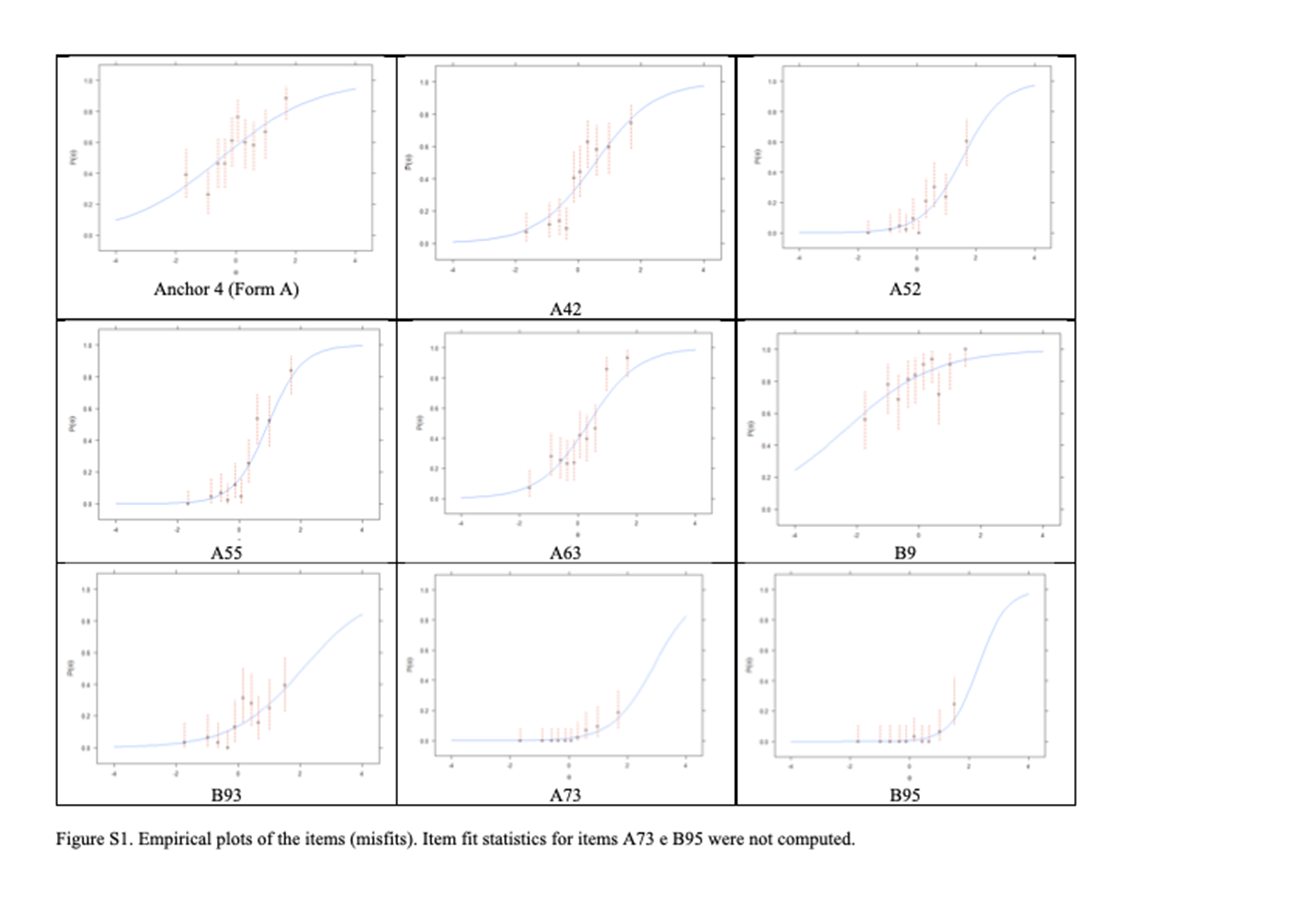

Supplement: Supplementary file 2 [file Image_1.tiff]
